# Discordance of retroperitoneal and thoracic histologic findings in patients with metastatic germ cell tumors at postchemotherapy residual tumor resection

**DOI:** 10.1186/s12957-024-03467-6

**Published:** 2024-07-17

**Authors:** Yue Che, Carolin Wöltjen, Achim Lusch, Christian Winter, Stephan Trainer, Moritz Schirren, Stefan Sponholz, Wolfram Trudo Knoefel, Peter Albers, Andreas Hiester

**Affiliations:** 1 Department of Urology, Medical Faculty, University of Duesseldorf, Heinrich- Heine-University, Duesseldorf, Germany; 2 Department of Thoracic Surgery, Agaplesion Markus Hospital Frankfurt, Frankfurt, Germany; 3 Division of Thoracic Surgery, Department of General and Visceral Surgery, Medical Faculty, University of Duesseldorf, Heinrich-Heine-University, Duesseldorf, Germany

**Keywords:** Germ cell cancer, Retroperitoneal surgery, Thoracic surgery, Postchemotherapy

## Abstract

**Introduction and objectives:**

Postchemotherapy residual tumor resection (PC-RTR) is an important part of the multimodal treatment for patients with metastatic germ cell tumors. Simultaneous retroperitoneal and thoracic metastases often require consecutive surgical procedures. This study analyzes the histologic findings after abdominal and thoracic surgery in order to tailor the sequence and intensity of surgery.

**Patients and methods:**

From a total of 671 PC-RTRs from 2008 to 2021 we analyzed 50 patients with stage III non-seminomatous germ cell tumor (NSGCT) who had undergone both retroperitoneal and thoracic postchemotherapy residual tumor resection after first-line and salvage chemotherapy.

**Results:**

All patients included had stage III NSGCT. 39 and 11 patients received first-line and salvage chemotherapy, respectively. 45 (90%) patients received retroperitoneal resection first, followed by thoracic surgery. Three patients (6%) underwent thoracic surgery before retroperitoneal surgery and two patients (4%) underwent simultaneous surgery. Overall, the histology of retroperitoneal and thoracic specimens was discordant in 23% of cases. After first-line chemotherapy, of fourteen patients with necrosis in retroperitoneal histology, four patients had vital carcinoma in lung histology. In patients with teratoma in the retroperitoneum, the thoracic findings were concordant in most cases (78%). When teratomatous elements were also present in the orchiectomy specimen, concordance was 100%. After salvage chemotherapy, the discordance rate was 55%.

**Conclusion:**

The data presented in this study underline that retroperitoneal residual masses with necrosis cannot reliably predict histologic findings of thoracic specimens. Patients with teratoma in the retroperitoneum have a high likelihood of teratoma in the thoracic specimen.

## Patient summary

In this report we the compared the findings of metastasic testicular cancer patients who received thoracic and retroperitoneal surgery. We concluded that the findings of one location cannot entirely predict the results of another location.

## Introduction

Postchemotherapy residual tumor resection (PC-RTR) plays an important role in the treatment of metastatic germ cell tumors (GCTs), although 40% of residual tumors have only necrotic tissue, 10% and 50% of residual tumors harbor viable cancer or teratoma, respectively [1]. Because residual tumor masses often involve other organs (kidney, lung, liver) or major vessels, PC-RTR is challenging and sometimes requires adjunctive vascular or organ surgery. Currently, the available diagnostic tools are still not reliable enough to predict residual tumor histology [2–4]. The morbidity of retroperitoneal surgery alone ranges from 7 to 30% with a mortality rate of 1% [5, 6]. In cases of resection of multiple tumor sites morbidity rates increase to over 35% [7, 8] and surgery must be carefully weighed against oncologic risk of recurrence. In cases of multiple residual tumor sites, current EAU guidelines recommend starting surgery at the site with the highest residual volume.

In our review of the literature, we found few studies comparing the correlation between retroperitoneal and thoracic histology [8–13]. Steyerberg et al. reported a high correlation (89%) between of necrotic findings in retroperitoneal lymph node dissection (RPLND) and lung resection [11]. However, other studies have reported discordance of 23–30% between different anatomical sites, mainly between retroperitoneum and chest [8, 10, 14]. Kesler et al. reported a discordance rate between retroperitoneal and thoracic pathology of 30% or more depending on the pathology category [12]. Schirren et al. reported a 20% discordance between the histology of both lungs when bilateral resection was performed. The discordance between mediastinal and lung histology was even higher at 28% [9]. On the other hand, Besse et al. described a discordance of only 5% between histologic findings of both lungs [10].

The aim of this study is to review our own data to analyze whether histology of one anatomical site can be used as a predictive tool.

## Patients and methods

Between 01/2008 and 03/2021, 671 patients underwent residual tumor resection for germ cell tumor (GCT) at our institution. We queried our database and identified 50 patients who underwent retroperitoneal and thoracic PC-RTR after first-line or salvage chemotherapy for metastatic non-seminomatous germ cell tumor (NSGCT). In a retrospective design we analyzed abdominal and thoracic histologies.

## Results

Patient characteristics are shown in Table 1. All patients had stage III NSGCT with retroperitoneal and thoracic metastases and underwent cisplatin-based chemotherapy followed by residual tumor resection. If not performed simultaneously, thoracic and retroperitoneal surgery was performed within 41 days (range 7–75 days) and no treatment was administered in between. Patients who received chemotherapy or radiotherapy between thoracic and abdominal resection were excluded from the study. Out of 50 patients, 10 had a good and intermediate prognosis, respectively. 30 patients had a poor prognosis according to IGCCCG.

**Table 1:** Patient’s characteristics

Patients	*n* = 50
**Age** (years, median, range)	34 (16–51)
**IGCCCG prognosis group**	
good intermediate poor	10 10 30
**Line of treatment**	
First line Salvage / further line	39 11
**First line chemotherapy**	
PEB / EP VIP Primary high dose (HD-VIP)	15 21 3
**Salvage chemotherapy**	
TI-CE TIP	10 1
**Timing of surgery**	
retroperitoneal surgery first thoracic surgery first simultaneous surgery	45 3 2
**Primary tumor**	
testicular tumor primary retroperitoneal / thoracic	47 3
**Tumor markers before surgery**,** first line group**	
HCG (mU/ml, median, range) AFP (µg/l, median, range)	0.3 (0–27.9) 3.5 (1.3–24.5)
**Tumor markers before surgery**,** salvage group**	
HCG (mU/ml, median, range) AFP (µg/l, median, range)	1.9 (0–8.8) 5.6 (2.5–2818)

39 patients underwent first line treatment with three patients undergoing primary high dose chemotherapy with cisplatin, ifosfamide and etoposide (VIP). 15 patients had bleomycin, etoposide and cisplatin (BEP) or etoposide and cisplatin (EP) and 21 patients had conventional dosed VIP.

Eleven patients underwent salvage chemotherapy, all of whom received the TI-CE regimen (paclitaxel [T] and ifosfamide [I] followed by high-dose carboplatin [C] and etoposide [E]). The order of resection site was based on tumor volume, beginning with the larger tumor. In 45 patients, retroperitoneal resection was performed before to thoracic surgery and in three cases, thoracic surgery was performed first. In two cases, thoracic and abdominal surgery were performed simultaneously. Median human chorionic gonadotropin (HCG) and alpha-fetoprotein (AFP) levels at the time of surgery were 0.3 mIU/ml (range: 0 -27.9) and 3.5 µg/l (range 1.3–24.5) in the first-line group and 1.9 (range: 0–8.8) and 5.6 (range: 2.5–2818) in the salvage group. One patient had a manifestly elevated AFP marker of 2818 µg/l after salvage chemotherapy.

We compared retroperitoneal and thoracic histologic reports of PC-RTR after first-line chemotherapy and salvage chemotherapy. All patients received retroperitoneal PC-RTR via open midline laparotomy. Of the thoracic surgeries, 41 patients received pulmonary PC-RTR only (wedge resections of the lung), 6 patients received extrapulmonary (mediastinal, retrocrural and/or cervical lymphadenectomy) PC-RTR only, and 4 patients received combined pulmonary and extrapulmonary PC-RTR.

Tumor size, detailed resection sites and adjunctive surgery are described in Table 2.

**Table 2:** Tumor size, resection sites and adjunctive surgery

	Retroperitoneal surgery *n* = 50	
**Prechemotherapy tumor size (mean)**	86 mm (range 7–350 mm)	
**Postchemotherapy tumour size (mean)**	72 mm (range 6–400 mm)	
**Adjunctive surgery**	15 (30%)	nephrectomy 5 patients (10%) aortic replacement 2 patients (4%) resection IVC 2 patients (4%) vertebral replacement 2 patients (4%) liver biopsy/resection 5 patients (10%) bowel resection 4 patients (8%)
	**Thoracic surgery*n*** = **50**	
**Prechemotherapy tumor size (mean)**	33 mm (range 9–110 mm)	
**Postchemotherapy tumour size (mean)**	27 mm (range 7–80 mm)	
**Resection site**		
**pulmonary** left lung right lung both sides	45 (88%) 33 (65%) 35 (69%) 24 (47%)	
**Extrapulmonary** + left lung + right lung + both sides Exclusive (only extrapulmonary resection)	10 (20%) 2 (4%) 1 (2%) 1 (2%) 6 (12%)	

### Histological outcomes

Histopathologic results after chemotherapy often contain mixed components of vital carcinoma, teratoma, necrosis, fibrosis and tumor-free lymph nodes. For the sake of clarity, histopathologic results are simplified in this manuscript and only the “worst” pathology is reported in this order: vital carcinoma (including teratoma with somatic-type malignancy), teratoma, necrosis.

### PC-RTR after first-line chemotherapy, table 3

**Table 3:** Comparison of retroperitoneal and thoracic histologies after first-line chemotherapy (inclusion of pulmonary and extrapulmonary histologies). Discordances between retroperitoneal and thoracic findings were 23%

First-line *n* = 39		Thoracic (pulmonary and extrapulmonary)				
		necrosis	teratoma	vital tumor other than teratoma	total	discordance %
**Retroperitoneal**	necrosis	**10**	0	4	14	29%
	teratoma	5	**18**	0	23	22%
	vital tumor other than teratoma	0	0	**2**	2	0
	total	15	18	6	**39**	**23%**

39 patients underwent PC-RTR after first-line treatment. On retroperitoneal histology 23 patients had teratoma, two had vital carcinoma and 14 had necrosis. On thoracic histology 18 patients had teratoma, 6 had vital carcinoma and 15 had necrosis.

When comparing retroperitoneal and thoracic histologic findings, the results were discordant in 23% of the cases. The highest discordance rate (29%) was found in patients with necrosis on retroperitoneal histology. Among fourteen patients with necrosis on retroperitoneal histology, four patients had vital carcinoma on pulmonary histology, one had teratoma with somatic-type malignancy (sarcomatoid differentiation), one had embryonal carcinoma, and two had unspecified non-seminoma. The pulmonary histology specimens containing viable embryonal carcinoma or non-seminoma were mostly composed of necrosis and had a proportion of viable tumor cells of 1–5%. In patients with retroperitoneal teratoma, the thoracic findings were concordant in most cases (78%). In patients with teratomous components in the orchiectomy specimen and the retroperitoneum, all had teratomous findings in the thoracic histology. Two patients had vital carcinoma in the retroperitoneum, specifically teratoma with somatic-type malignancy, and had similar thoracic findings.

When comparing retroperitoneal and only extrapulmonary thoracic histologies, the findings were concordant in 100% of the patients, eight of these patients had teratoma and one had teratoma with somatic-type malignancy (liposarcoma).

### PC-RTR after salvage chemotherapy, table 4

**Table 4:** Comparison of retroperitoneal and thoracic histologies after salvage chemotherapy. In 55% of patients retroperitoneal and thoracic histology did not match

Salvage treatment *n* = 11		Thoracic (pulmonary and extrapulmonary)				
		necrosis	teratoma	vital tumor other than teratoma	total	discordance %
**Retroperitoneal**	necrosis	**4**	0	2	6	33%
	teratoma	2	**1**	0	3	67%
	vital tumor other than teratoma	1	1	**0**	2	100%
	total	7	2	2	**11**	**55%**

Of the 11 patients who underwent PC-RTR after salvage chemotherapy, six patients had discordant histologic findings. On retroperitoneal histology three patients had teratoma, two had vital carcinoma (yolk sac tumor and choriocarcinoma), and six had necrosis. In thoracic histology, two patients had teratoma, two had vital carcinoma (embryonal carcinoma and unspecified non-seminoma), and seven had necrosis. Teratoma with somatic-type malignancy was not observed in this group. Of note, all patients with vital carcinoma in the retroperitoneum did not have vital carcinoma in the thoracic histology and vice versa. Only the patient with yolk sac tumor in the retroperitoneum had a highly elevated AFP preoperatively. All the other patients had negative or slightly elevated tumor markers.

### Comparison of left and right sided pulmonary resection, table 5

**Table 5:** Comparison of histologies of left and right sided pulmonary resection. Overall, 24 patients received bilateral pulmonal resection after either first-line or salvage therapy. Overall, 13% of histologies between left and right sided resection differed

Histology		Right lung				
		necrosis	teratoma	vital tumor other than teratoma	total	discordance %
**Left lung**	necrosis	**12**	2	0	14	17%
	teratoma	0	**8**	0	8	0%
	vital tumor other than teratoma	1	0	**1**	2	50%
	total	13	10	1	**24**	**13%**

Twenty-four patients underwent bilateral pulmonary PC-RTR. Three cases (13%) had discordant histologies between the two sides. Two patients had teratoma on the right side and necrosis on the left side. One patient had vital carcinoma on the left side and necrosis on the right side.

## Discussion

This study confirms an important finding, namely, that we cannot fully rely on the predictive value of the histologic findings in one anatomic site for PC-RTR in another anatomic site.

Approximately 20% of testicular cancer patients are overtreated and, due to the excellent survival rates, even in metastatic stages, treatment-related toxicities are the main cause for morbidity in these patients [15–17]. The reduction of treatment-related toxicity should be a central measure in decision making. Thus, the idea of avoiding unnecessary surgery is appealing, especially when the histologic result of one metastatic site is predictive of another.

In the present study, we evaluated both first-line and salvage patients. Since the therapeutic options after salvage chemotherapy are limited, the indication to resect residual masses in this setting is imperative. The associated willingness to take surgical risks surgery is particularly important in the salvage setting and must therefore be evaluated separately from the first-line setting.

Several groups have described discordant histologic findings when resection is performed at multiple sites. In the present study, we report a histologic discordance rate between retroperitoneal and thoracic histology of 23% after first-line treatment and 55% after salvage treatment. These findings are consistent with large series describing discordance between histologic findings from different sites ranging from 29 to 46% [11, 14, 18]. However, in contrast to these previous studies, we cannot confirm the predictive value of necrotic tissue in the retroperitoneum for an identical histology in thoracic residual masses. Steyerberg and Hartman both described that in the case of presence of necrotic tissue in the retroperitoneum, the probability of necrotic tissue in pulmonary lesions could be up to 90% [11, 14]. In our cohort, patients with necrosis only on retroperitoneal histology had a discordance with thoracic histology of 29% for first-line patients and 33% for salvage patients.

Complete concordance was found when comparing between histologic findings from retroperitoneal and extrapulmonary thoracic sites, all of which contained teratomous elements. This is most likely be due to the common lymphogenic spread of retroperitoneal and thoracic lymphatic metastases.

Twenty-four patients underwent bilateral lung resection. In this cohort, the discordance of histologic findings on both sides was 13%. Already in 2009 Besse et al. described that in case of bilateral lung metastasis and necrosis on one side, the probability of the same histology on the other side is up to 95% [10]. In contrast, to these findings Schirren et al. described a discordance of histologic findings between left and right lung of 20% [9].

An important finding that could be used to predict treatment outcome und thus guide further decision making is the fact that all patients with teratomous findings at orchiectomy and retroperitoneal resection also had teratoma on thoracic histology. The current guideline recommendation also requires resection of the thoracic residues in the case of teratomas in the retroperitoneum [19].

### Limitations

Although this study was conducted in one of the largest germ cell tumor referral centers in Germany, the cohort size presented is still small and did not allow for an analysis with statistically sound conclusions. In addition, the rarity of the disease limits efforts such as this one to a retrospective analysis. Furthermore, patients with residual retroperitoneal and thoracic disease who did not undergo surgery were not evaluated in this study. In our center, indications for resection of residual tumors are generally residual masses larger than 1 cm. However, several factors may influence the decision to proceed with surgery. A high degree of tumor shrinkage after chemotherapy, a high proportion of seminoma or choriocarcinoma in the primary histology (which would typically contain only necrosis after chemotherapy), or poor patient health status may be factors that weigh in favor of surveillance. The data presented in this study are subject to a selection bias because all patients included in this study were selected for surgery at retroperitoneal and thoracic sites.

## Conclusion

Based on our analysis, necrosis in the retroperitoneal specimen does not exclude vital carcinoma at another anatomical site. Therefore, we still recommend the resection of relevant residual thoracic metastases, even if the retroperitoneal specimen was only necrotic. If the retroperitoneal specimen contained teratoma, the thoracic residual masses are very likely to contain teratoma as well. Reliable prediction between retroperitoneal and extrapulmonary thoracic metastases is possible.

## Data Availability

No datasets were generated or analysed during the current study.

## Abbreviations

PC-RTR: Postchemotherapy residual tumor resection
GCT: Germ cell tumor
EAU: European Association of Urology
NSGCT: Non seminomatous germ cell tumor
IGCCCG: International Germ Cell Cancer Cooperative Group
VIP: Cisplatin, ifosfamide and etoposide
BEP: Bleomycin, etoposide and cisplatin
EP: Etoposide and cisplatin
TI-CE: Paclitaxel and ifosfamide - carboplatin and etoposide
HCG: Human chorion gonadotropin
AFP: Alpha fetoprotein
